# Persistent Mu-Opioid Receptor Dysregulation in a Pain-Facilitatory Brain Region Reinstates Hyperalgesia After Resolution of Opioid-Induced Hyperalgesia

**DOI:** 10.3390/ph19050695

**Published:** 2026-04-28

**Authors:** Marília Sousa, Ana Rita Costa, Isaura Tavares, Isabel Martins

**Affiliations:** 1 Departamento de Biomedicina, Faculdade de Medicina, Universidade do Porto, 4200-319 Porto, Portugal; 2 i3S—Instituto de Investigação e Inovação em Saúde, Universidade do Porto, 4200-135 Porto, Portugal; 3 IBMC—Instituto de Biologia Molecular e Celular, Universidade do Porto, 4200-135 Porto, Portugal; 4 Institute of Pharmacology and Toxicology, University of Zurich, 8057 Zurich, Switzerland; 5 Science for Life Laboratory, Department of Biochemistry and Biophysics, Stockholm University, 114 19 Stockholm, Sweden

**Keywords:** opioid-induced hyperalgesia, descending pain facilitation, μ-opioid receptor, morphine, latent sensitization

## Abstract

**Background/Objectives:** Opioids paradoxically induce hypersensitivity that typically resolves after discontinuation. Despite normalization of sensitivity, the nociceptive system may remain primed. Descending pathways contribute to opioid-induced hyperalgesia (OIH), and the following latently maintained sensitization. The mechanisms underlying the latter vulnerability remain unknown. We previously showed that the dorsal reticular nucleus (DRt), a key brainstem area involved in pain facilitation, drives OIH through a maladaptation in μ-opioid receptor (MOR) signaling. Whether cellular alterations in the DRt persist after opioids are discontinued and hyperalgesia resolves is unknown. Here, we investigated the long-term effects of morphine on DRt MOR expression, signaling, and function after hyperalgesia has been resolved. **Methods:** Male Wistar rats received morphine for 7 days either subcutaneously or continuously. Nociceptive sensitivity was evaluated by von Frey and hot-plate tests. MOR and phosphorylated CREB (pCREB) expression in the DRt were quantified during OIH and after hyperalgesia resolution. In the post-OIH phase, we evaluated the effects of MOR activation by DAMGO at DRt, postoperative pain behavior, and systemic morphine dose–response curves. **Results:** Both morphine regimens induced hypersensitivity that subsided within two weeks. MOR expression in the DRt increased during OIH and normalized in the post-OIH phase, whereas pCREB levels remained elevated in both phases. In the post-OIH phase, DAMGO microinjection at the DRt reinstated robust hypersensitivity and systemic morphine showed reduced antiallodynic potency in postoperative pain. **Conclusions:** Chronic morphine leaves a lasting molecular imprint in the DRt, sustaining excitatory MOR signaling that can reinstate hyperalgesia and likely diminishes later opioid analgesic efficacy.

## 1. Introduction

The repeated administration of opioids can produce several deleterious effects, including a paradoxical increase in pain sensitivity known as opioid-induced hyperalgesia (OIH). This phenomenon is well established in animal models [1] and has also been demonstrated in human studies [2]. The heightened pain sensitivity characteristic of OIH has been shown to subside following opioid cessation [3,4,5,6,7]. However, even after nociceptive hypersensitivity resolves, the prior opioid exposure has been shown to prime the nociceptive system, rendering it more vulnerable to subsequent pain or injury. In humans, this priming effect is evident across multiple cohorts, including former opioid-dependent individuals maintained on methadone, surgical patients receiving perioperative opioids, and healthy volunteers exposed to acute opioid administration [8,9]. Studies in these populations consistently demonstrate persistent nociceptive sensitization long after the original opioid exposure, reflected by higher postoperative pain intensity scores [8] and by reactivation of pain at previous injury sites [9]. In animal models, this priming effect has been shown to be triggered by heroin [3], morphine [4,5,6,7], fentanyl [10,11,12], and remifentanil [13]. The animals develop a chronic latent hyperalgesia that silently persists after behavioral signs of pain resolution and this state has been designated as latent pain sensitization [3,14,15]. Chronic inflammatory and neuropathic pain develop a similar state of latent sensitization, after healing of the primary insult [14,15]. Importantly, many of the biological changes that promote sensitization after injury also occur with opioid exposure, and these effects tend to reinforce one another [14,15]. Mechanistically, the phenomenon of opioid-induced latent sensitization involves activation of N-methyl-D-aspartate receptors (NMDARs) [11,13], and µ-opioid receptors (MOR) [16] or κ-opioid receptors (KOR) [13]. At the cellular level, opioids have been shown to prime primary afferent nociceptors, increasing their responsiveness to subsequent pronociceptive agents, a well-established phenomenon termed hyperalgesic priming [17,18,19]. Latent sensitization is also maintained through central mechanisms, particularly those involving descending pain modulatory circuits [12], but the underlying molecular mechanisms remain unknown. The persistent engagement of descending facilitatory circuits observed during OIH [20], together with the enhanced sensitization of these circuits in chronic pain [1,21], highlights the importance of investigating the central mechanisms underlying opioid-induced sensitization.

The Dorsal reticular nucleus (DRt) is a medullary region that plays a central role in the descending facilitation of both acute and chronic pain [1,21,22,23]. It has been shown that the local opioidergic inhibitory circuit constitutes a buffer to counter DRt facilitation in acute pain [24,25]. During chronic pain, however, the opioidergic signaling within the DRt undergoes significant neural adaptations and these changes reduce the effectiveness of local opioid-mediated inhibition and enhance the facilitatory output of the DRt, thereby contributing to the persistence and amplification of pain [26,27]. We also found that enhanced descending facilitation from the DRt constitutes a key driver of OIH [20]. We showed that chronic morphine exposure induces a functional switch in MOR signaling within the DRt from inhibitory to excitatory G-protein coupling [20]. Our findings indicate that this MOR signaling switch constitutes a key molecular event underlying the development of OIH. Here aim to explore whether the DRt also plays a critical role in maintaining central opioid-dependent sensitization. For that, we first established the time course of hypersensitivity onset and resolution in morphine-treated animals. We then examined MOR expression and signaling in the DRt at the time point corresponding to both hypersensitivity onset and resolution. To assess changes in MOR signaling, we analyzed the labeling of phosphorylated cAMP response element-binding protein (pCREB), a downstream marker of the excitatory signaling pathway [20,28]. At the time of resolution of hyperalgesia, we further evaluated the behavioral effects of the MOR activation at the DRt by the MOR agonist DAMGO. Finally, we determined the efficacy of morphine in treating post-operative pain in animals previously exposed to chronic morphine following complete reversal of OIH.

## 2. Results

### 2.1. Time Course Analysis of Nociceptive Sensitivity Following Resolution of Hyperalgesia

We evaluated nociceptive sensitivity after cessation of a 7-day morphine regimen administered either via continuous infusion or subcutaneously. These two morphine administration regimens represent two common and validated models of OIH [29,30].

**Figure 1:**
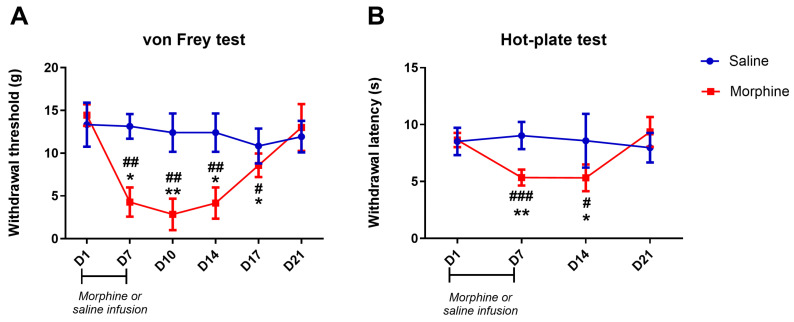
Time course effects of upon cessation of morphine or saline infusion with osmotic mini-pumps on mechanical sensitivity tested by the von Frey test (**A**) and on heat hypersensitivity tested by the hot-plate test (**B**). Data are presented as mean ± SD (saline–blue line *n* = 6; morphine–red line *n* = 5). * *p* < 0.05; ** *p* < 0.01 vs. day 1; # *p* < 0.05; ## *p* < 0.01; ### *p* < 0.001 vs. saline-treated-animals. D: Day.

Figure 1 shows the time course evaluation of basal sensitivity in morphine- and saline-treated animals administered continuously via osmotic minipumps. The sensitivity was assessed by the von Frey and hot-plate tests on day 1, prior to minipump implantation, and on day 7, prior to minipump removal. Testing continued after removal of the mini-pumps, terminating the delivery of morphine, on days 10, 14, 17, and 21. Testing by the hot-plate test after termination of the infusion was only performed on days 14 and 21, to avoid additional heat-induced sensitization of the paw. Morphine-infused animals developed mechanical hypersensitivity as shown by decreased withdrawal thresholds on day 7 compared to day 1 (before the implantation of mini-pumps; *p* = 0.011; Figure 1A) and saline-infused animals (*p* = 0.002; Figure 1A), as we have previously reported [20]. On days 10, 14, and 17 (after removal of the mini-pumps) the animals continued to show a decrease in withdrawal thresholds compared to baseline and saline (Figure 1A). On day 21, no significant differences were observed demonstrating a complete reversal of morphine-induced mechanical hypersensitivity (Figure 1A). In the hot-plate test, the animals infused with morphine developed thermal hypersensitivity as shown by a significant decrease in the withdrawal latency to heat on day 7 compared to day 1 (*p* = 0.001; Figure 1B) and saline (*p* < 0.001; Figure 1B), in a manner similar to our previous reports [20]. On day 14, morphine-treated animals continued to show a decrease in withdrawals compared to day 1 and saline-treated animals (*p* < 0.05; Figure 1B). On day 21, withdrawals of morphine-treated animals were no longer different from day 1 and saline-treated animals, showing a complete reversal of heat hypersensitivity at this timing (Figure 1B). No significant differences were observed between saline- and morphine-infused animals on day 1 in both tests. The withdrawal thresholds and latencies of saline-infused animals were not significantly altered throughout the time-course analysis.

**Figure 2:**
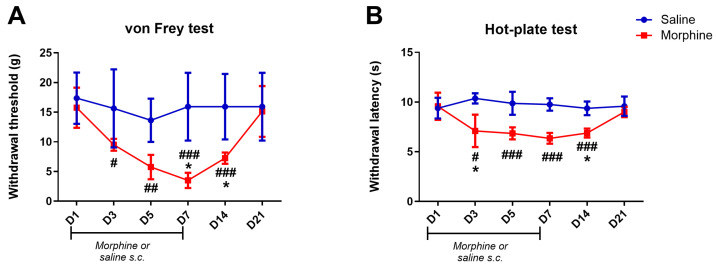
Time course effects of subcutaneous administration of morphine or saline and upon treatment cessation on mechanical sensitivity tested by the von Frey test (**A**) and on heat hypersensitivity tested by the hot-plate test (**B**). Data reported was measured before the morning injection of morphine. Data are presented as mean ± SD (saline–blue line *n* = 7; morphine–red line *n* = 4). * *p* < 0.05 vs. D1; # *p* < 0.05; ## *p* < 0.01; ### *p* < 0.001 vs. saline-treated-animals. D: Day.

Figure 2 shows the time course of basal sensitivity in morphine- and saline-treated animals under the subcutaneous regimen. Mechanical and thermal sensitivities were respectively evaluated by the von Frey and hot-plate tests performed in the morning prior to injections on days 1, 3, 5, and 7. Unlike in the experimental groups treated with osmotic minipumps, multiple intermediate time points were assessed before day 7, as this was the first time this model of OIH was implemented in our laboratory. Testing continued after cessation of treatment on days 14 and 21, corresponding to one and two weeks after cessation of treatment. These post-treatment time points were selected based on the results obtained in animals treated with osmotic minipumps (Figure 1), which were sufficient to capture the resolution phase of the hyperalgesic response. The animals treated with morphine, developed mechanical and heat hypersensitivity, as previously showed [29]. In the von Frey test, morphine-treated animals showed a significant decrease in withdrawal thresholds on at days 3, 5 and 7 compared to saline (Figure 2A). On day 7, withdrawals were also significantly lower compared to before the first injections on day 1 (*p* = 0.031; Figure 2A). After treatment cessation on day 14, withdrawal thresholds in morphine-treated animals remained statistically lower than in saline-treated animals (*p* < 0.001; Figure 2A) and day 1 (*p* = 0.031; Figure 2A). On day 21, morphine-treated animals showed no significant differences compared to saline baseline and day 1, demonstrating a complete reversal of morphine-induced mechanical hypersensitivity at this timing (Figure 2A). No significant differences were observed between morphine- and saline-treated animals on day 1. Withdrawal thresholds of saline-treated animals were not significantly altered throughout the time-course.

In the hot-plate test, morphine-treated animals showed a significant decrease in paw-withdrawal latencies on days 3, 5, and 7 compared to saline (Figure 2B). On day 3, withdrawals were also significantly lower compared to day 1 (*p* = 0.022; Figure 2B). After treatment cessation on day 14, withdrawal latencies of morphine-treated animals were still significantly lower than in saline-treated animals (*p* < 0.001; Figure 2B) and, marginally, than day 1 (*p* = 0.051; Figure 2B). On day 21, withdrawal latencies of the morphine group were no longer significantly different from saline-treated animals and day 1, indicating a complete reversal of morphine-induced heat hypersensitivity at this timing (Figure 2B). No significant differences were observed between morphine- and saline-treated animals at day 1. Withdrawal thresholds of saline-treated animals were not significantly altered throughout the time-course.

**Figure 3:**
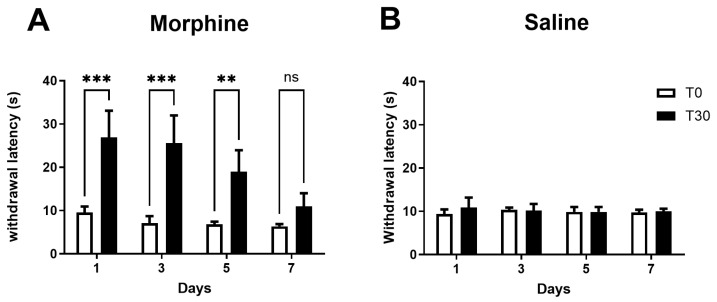
Antinociception assessed by the hot-plate test. The animals were injected twice daily (at 9 a.m. and 6 p.m.) for seven days. To assess the acute antinociceptive effects of morphine, the hot-plate test was performed before (T0) and 30 min (T30) after the first injection of morphine (**A**) or saline (**B**), in the morning, at Days 1, 3, 5, and 7. Data are presented as mean ± SD (saline-treated animals, *n* = 7; morphine-treated animals, *n* = 4); ** *p* < 0.01, *** *p* < 0.001; ns: non-significant.

We also evaluated the antinociceptive effects morphine (Figure 3A) and saline (Figure 3B) on Days 1, 3, 5, and 7 by the hot-plate test conducted before and 30 min after the first daily injections (in the morning). Morphine significantly increased withdrawal latencies compared to before injection on days 1, 3, and 5 (Figure 3A), indicating that morphine induced antinociceptive effects in these days. On Day 7, morphine did not produce any significant change compared to before the injection (Figure 3A). The injection of saline produced no significant effects (Figure 3B).

### 2.2. Analysis of MOR Signaling at a Pain Facilitatory Area After Resolution of Hyperalgesia

**Figure 4:**
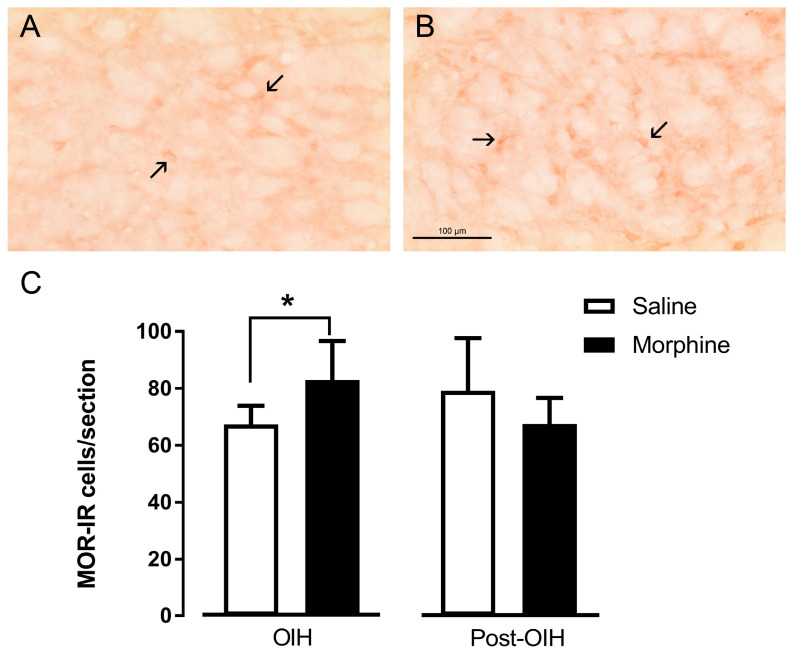
MOR expression at the DRt at 7 days after continuous infusion of morphine (OIH group) and two weeks after morphine cessation (post-OIH group). Control animals were infused with saline. MOR-immunoreactive (MOR-IR) cells were quantified in five sections per animal, spanning the rostrocaudal extent of the DRt. Representative photomicrographs of MOR labeling at the DRt taken from animals after 7 days of treatment with saline (**A**) or morphine ((**B**); OIH group). Typical MOR-IR labelling is marked by arrows in (**A**,**B**). Scale bar in (**B**): 100 μm ((**A**) is at the same magnification). Data in (**C**) are presented as mean ± SD (OIH group: saline or morphine *n* = 6 each; Post-OIH group: saline or morphine *n* = 6 each). * *p* < 0.05.

**Figure 5:**
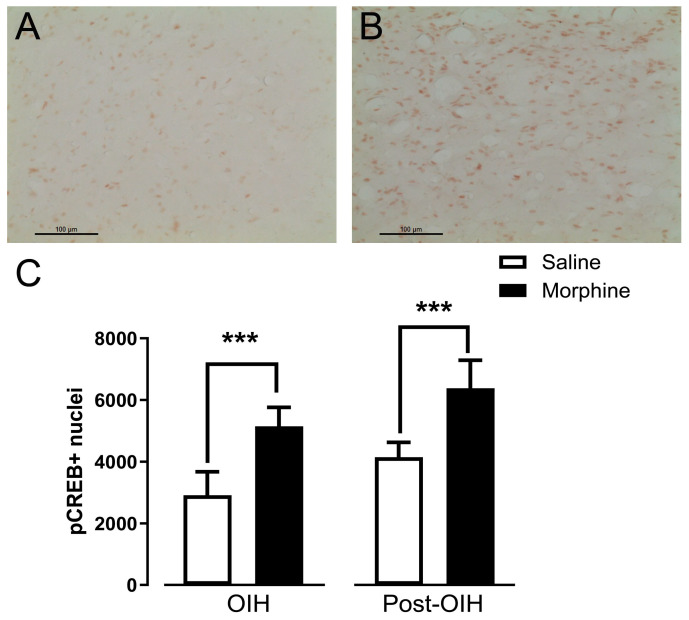
pCREB expression at the DRt at 7 days after continuous infusion of morphine (OIH group) and two weeks after morphine cessation (post-OIH group). Control animals were infused with saline. pCREB+ nuclei were quantified in five sections per animal, spanning the rostrocaudal extent of the DRt. Representative photomicrographs of pCREB labeling at the DRt taken from animals after 7 days of treatment with saline (**A**) or morphine ((**B**); post-OIH group). Scale bar in (**A**,**B**): 100 μm. Data in (**C**) are presented as mean ± SD (OIH group: saline or morphine *n* = 6 each; Post-OIH group: saline or morphine *n* = 6 each). *** *p* < 0.001.

We evaluated the effects of morphine on MOR signaling in the DRt through the quantification of the immunolabeling of canonical MOR and pCREB, on two different timings: 7 days after continuous infusion of morphine or saline (corresponding to animals from the OIH group in Figure 4 and Figure 5), and two weeks after treatment cessation and hyperalgesia resolution (corresponding to animals from the post-OIH group in Figure 4 and Figure 5).

The analysis of MOR-IR cells showed a significant increase in the number of MOR-IR cells at the DRt of morphine-treated animals compared with saline treatment (*p* = 0.031; Figure 4A–C). In the Post-OIH group, no significant differences were observed between animals previously treated with morphine and saline (Figure 4C).

The numbers of pCREB + nuclei at the DRt of morphine-treated animals was significantly higher than in saline-treated animals both in the OIH and post-OIH groups (*p* < 0.001; Figure 5A–C).

**Figure 6:**
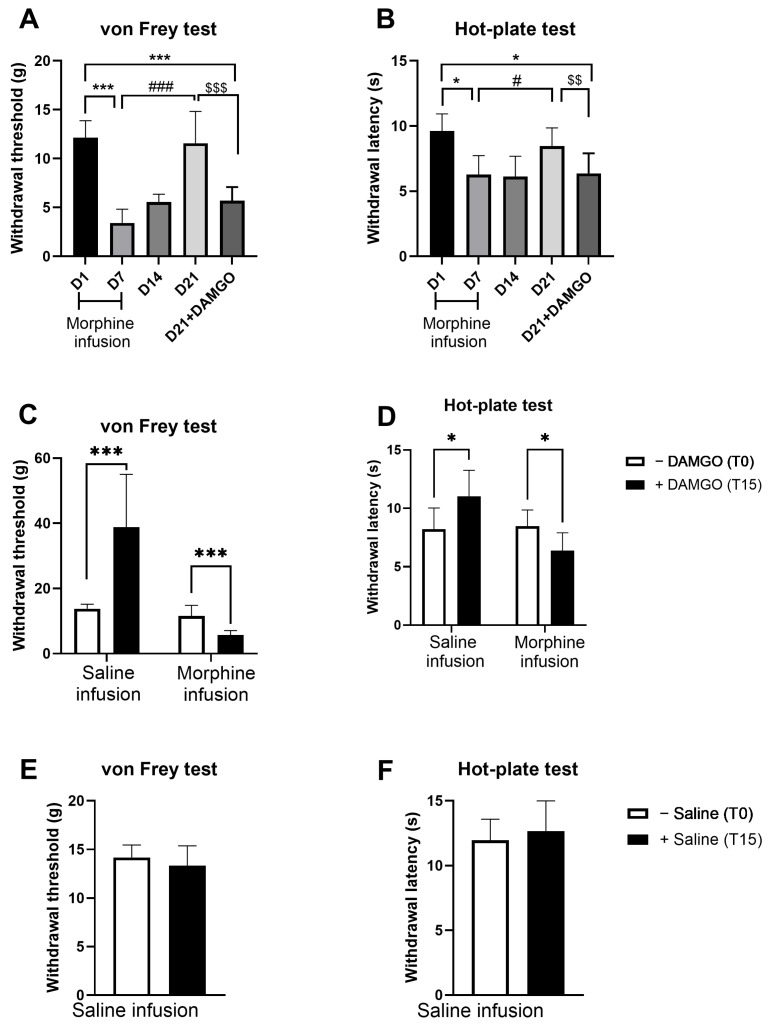
Effects of MOR activation by agonist DAMGO on the nociceptive behavior after resolution of hyperalgesia. Mechanical sensitivity was tested by the von Frey test (**A**,**C**,**E**) and heat sensitivity was tested by the hot-plate test (**B**,**D**,**F**). Time course analysis of mechanical (**A**) and heat sensitivity (**B**) of morphine-infused animals on days 1, 7, 14, 21, and on day 21 after DAMGO injection. Mechanical and thermal sensitivity on day 21, before (T0), and 15 min (T15) after DAMGO (**C**,**D**) or the saline vehicle (**E**,**F**) injection into the DRt. Data are presented as mean ± SD (DAMGO injections: saline-infused animals *n* = 4; morphine-infused animals *n* = 7; saline vehicle injections on saline-infused animals *n* = 6). * *p* < 0.05; *** *p* < 0.001; # *p* < 0.05; ### *p* < 0.001; $$ *p* < 0.01; $$$ *p* < 0.001. D: Day.

We also determined the effects of MOR activation by the MOR agonist DAMGO on the nociceptive behavior after resolution of hyperalgesia. For that, we used a group of animals previously infused with either saline or morphine for 7 days. On day 21 (corresponding to two weeks after treatment cessation), DAMGO was microinjected through a cannula implanted above the DRt. Figure 6A,B shows the time course analysis of nociceptive behavior in morphine-infused animals, assessed by the von Frey and hot-plate tests. On day 7, morphine-treated animals developed mechanical and thermal hypersensitivity, shown by the significant drop of mechanical withdrawal thresholds (D7 vs. D1 *p* < 0.001; Figure 6A) and withdrawal latencies (D7 vs. D1: *p* < 0.02; Figure 6B). This hypersensitivity completely vanished by day 21. At this time point, mechanical withdrawal thresholds increased significantly compared to D7 (*p* < 0.001, Figure 6A), returning to baseline levels observed on day 1 (Figure 6A). Similarly, withdrawal latencies also increased significantly compared to D7 (*p* = 0.012, Figure 6A), returning to baseline levels observed at day 1 (Figure 6B). The injection of DAMGO reinstated both mechanical and thermal hypersensitivity. DAMGO significantly reduced withdrawal thresholds and withdrawal latencies compared both to pre-injection values (day 21) and day 1 (Figure 6A,B).

Figure 6C,D shows the comparison of DAMGO effects between morphine- and saline-infused animals. DAMGO injection, produced opposite effects: analgesia in saline-infused animals and hypersensitivity in morphine-infused animals. In the von Frey test, withdrawal thresholds increased significantly after DAMGO injection compared to before injection in saline-infused animals, whereas thresholds decreased significantly in morphine-infused animals (*p* < 0.001; Figure 6C). Similarly, in the hot-plate test, withdrawal latencies increased significantly after DAMGO injection in saline-infused animals, while latencies decreased significantly in morphine-infused animals (*p* < 0.05; Figure 6D). The injection of the vehicle (saline) into the DRt of saline-infused animals produced no significant effects (Figure 6E,F).

### 2.3. Study of Post-Operative Pain After Resolution of Hyperalgesia

**Figure 7:**
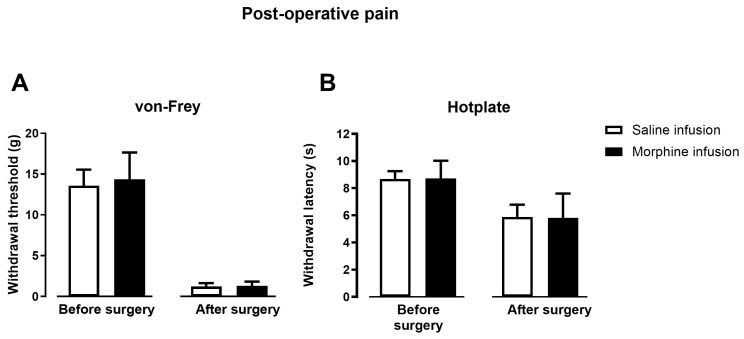
Nociceptive sensitivity before and after induction of post-operative pain in animals that had previously received a 7-day infusion of saline or morphine. The von Frey (**A**) and hot-plate tests (**B**) were conducted on day 21, corresponding to two weeks after termination of the infusion. Data are presented as mean ± SD (Saline-infusion *n* = 7; Morphine-infusion *n* = 7).

We established a post-operative pain model in animals that had previously received a 7-day infusion of either saline- (*n* = 7) and morphine- (*n* = 7). The model was induced on day 21, corresponding to two weeks after termination of the infusion), following complete reversal of OIH. We initially evaluated the impact of resolved OIH on the intensity of post-operative pain by the von Frey and hot-plate tests. After induction of post-operative pain there was a significant decrease in withdrawal thresholds in the von Frey test (*p* < 0.001; Figure 7A) and latencies in the hot-plate test (*p* < 0.001; Figure 7B), indicating that the surgery induced hyperalgesia. However, prior exposure to morphine did not exacerbate hypersensitivity, as no significant differences were found between saline- and morphine-infused animals in both tests (Figure 7).

**Figure 8:**
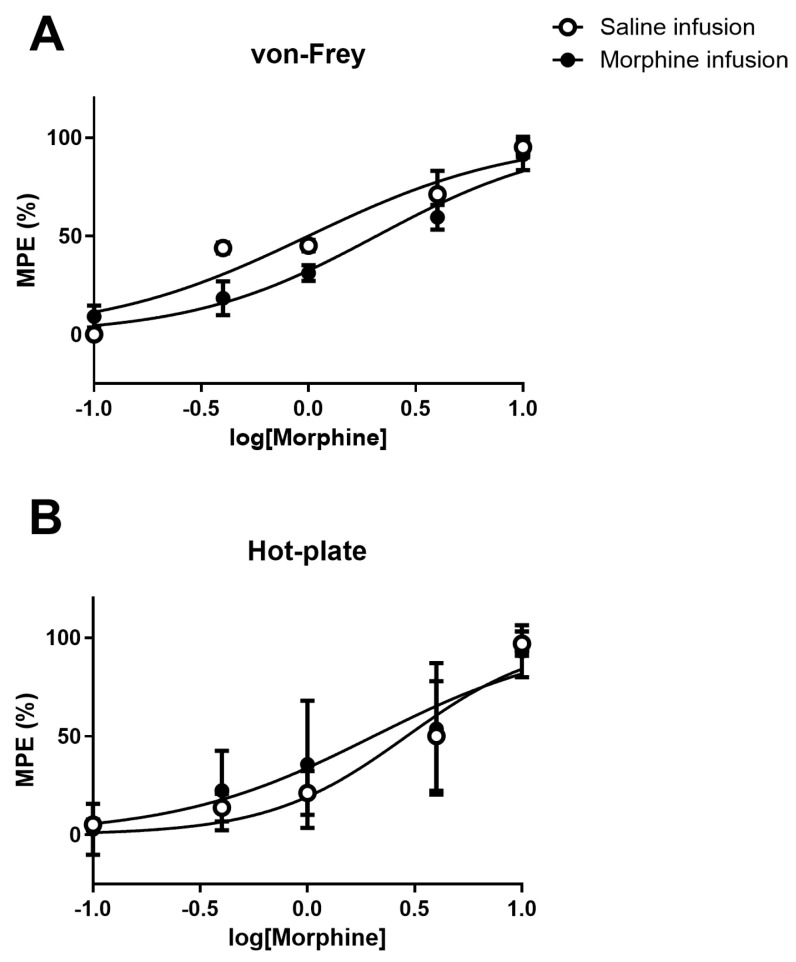
Analgesic potency of systemic morphine on post-operative pain assessed by the von Frey (**A**) and hot-plate tests (**B**) in animals that had previously received a 7-day infusion of saline or morphine. Incrementing doses of morphine (0.1, 1, 4, and 10 mg/Kg) were administered on day 21, corresponding to two weeks after termination of the infusion, following complete reversal of OIH. Cumulative dose response curves of systemic morphine were plotted as percentage of maximum possible effect (% MPE) and fitted by nonlinear regression. Data are presented as mean MPE ± SD (Saline-infusion *n* = 7; Morphine-infusion *n* = 7).

We next tested the effects of cumulative doses of systemic morphine on the von Frey and hot-plate tests. Data were plotted as percentage of maximum possible effect (% MPE) and fitted by non-linear regression (Figure 8A,B). The analysis of data obtained in the von Frey test, showed that the incrementing doses of morphine significantly increased the MPE in both groups (*p* < 0.001) and that the MPE of morphine was significantly higher in animals previously treated with saline (*p* = 0.004). The morphine dose that produced 50% of the MPE, i.e., the ED50 in animals previously infused with morphine was shifted to the right 2-fold. The ED50 in the saline-treated group was 0.997 mg/kg (95% CI: 0.786–1.265 mg/kg; Figure 8A) and the ED50 in morphine animals was 2.074 mg/kg (95% CI: 1.745–2.465 mg/kg; Figure 8A).

The analysis of data obtained in the hot-plate test also showed that the incrementing doses of morphine significantly increased the MPE in both groups (*p* < 0.001), but no differences were found between saline- and morphine- treated animals (*p* = 0.537). The morphine dose that produced 50% of the MPE, i.e., the ED50 in the saline-treated group was 2.906 mg/kg (95% CI: 2.184–3.866 mg/kg; Figure 8B), while in morphine animals it was 2.036 mg/kg (95% CI: 1.212–3.418 mg/kg; Figure 8B).

## 3. Discussion

We previously showed that a 7-day chronic morphine treatment producing paradoxical hyperalgesia entailed a switch of MOR signaling at the DRt, a brainstem area involved in descending pain facilitation. Specifically, we showed a shift of MOR signaling from inhibitory to excitatory transforming this pain facilitatory area into a driver of OIH [20]. To investigate whether this maladaptive plasticity persists after treatment cessation and hyperalgesia has subsided, we treated naïve rats with morphine for seven days and performed several molecular and behavioral studies two weeks after morphine cessation, once hyperalgesia had subsided. We observed intracellular alterations in MOR signaling at the DRt, indicating the persistence of excitatory MOR signaling. We also found that the administration of the MOR agonist DAMGO, at the DRt, reinstated hyperalgesia. Finally, we showed that systemic morphine exhibited decreased antiallodynic potency in incisional pain in animals previously exposed to morphine. These findings indicate that despite hyperalgesia subsides, MOR signaling at a pain facilitatory area of the brain remains inherently altered. The persistence of this maladaptation predisposes subsequent activation of MOR to exacerbate descending facilitation rather than suppressing it, likely contributing to the decline of morphine’s therapeutic potency.

Our behavioral studies show that a 7-day morphine treatment induced mechanical and heat hypersensitivity and that upon morphine cessation, nociceptive thresholds gradually returned to pre-treatment values within approximately two weeks. The behavioral pattern observed during morphine treatment is consistent with the classical development of OIH, in a manner similar to previous reports [20,30]. The normalization of nociceptive thresholds upon morphine cessation is also similar to previous reports [4,5,6,7]. Interestingly, we found that at a timepoint of normalization of the nociceptive thresholds, i.e., in the post-OIH phase, microinjection of DAMGO into the DRt, reinstated robust hyperalgesia. The development of hyperalgesia followed by normalization of nociceptive thresholds and subsequent re-instatement of hyperalgesia suggests the installation of the phenomenon of latent pain sensitization, a phenomenon that occurs after both opioid exposure and inflammatory or neuropathic injuries [14,15]. A defining characteristic of opioid induced latent sensitization is the reinstatement of hyperalgesia by a subsequent pharmacological challenge [3,7] or by an external stimulus such as stress [11], unmasking the underlying sensitized state of the nociceptive system. Our behavioral studies further reveal that the temporal profile for the onset and resolution of hyperalgesia was similar for the two morphine regimens tested, i.e., repeated injections and continuous infusion. The congruent timelines across administration methods suggest that morphine sensitizes the nociceptive system via shared underlying mechanisms, an insight highly relevant to clinical practice, where morphine may be administered intermittently or continuously for the management of severe pain [2], and thus both regimens may predispose patients to the development of latent sensitization.

We showed that pCREB levels remained elevated during both the OIH- and post-OIH phases. The upregulation of pCREB is one of the hallmarks of a switch of signaling of MOR from inhibitory to excitatory [28,31,32]. Consistent with this, we previously demonstrated that increased pCREB expression in the DRt was associated with a functional shift in MOR signaling from inhibitory to excitatory, driven by the preferential coupling of MOR to Gs proteins rather than Gi proteins [20]. We further showed that this shift of MOR signaling turned the DRt into a driver of OIH [20]. The fact that pCREB expression remains upregulated after morphine cessation, in the post-OIH phase, suggests that a “molecular memory” of altered MOR signaling persists beyond the resolution of hyperalgesia. Further supporting this, the activation of MOR with DAMGO during the post-OIH phase, when nociceptive thresholds have normalized, reinstates hyperalgesia. Thus, prior exposure to opioids induces a molecular imprint of altered MOR function at a pain facilitatory area that can be reactivated by later pharmacological challenges. Supporting the functional relevance of this latent state, pharmacological activation of MOR with DAMGO during the post-OIH phase—when nociceptive thresholds have returned to baseline—was sufficient to reinstate hyperalgesia. Together, these observations indicate that prior opioid exposure leaves a persistent molecular imprint consistent with altered MOR signaling in a pain-facilitatory region, which can be re-engaged by subsequent opioid challenge. Beyond serving as a neural correlate of the MOR signaling switch, pCREB likely contributes to the maintenance of a latent sensitized state within the DRt. This interpretation is consistent with extensive evidence identifying pCREB as a key molecular mediator of long-term nociceptive memory and persistent pain-related synaptic plasticity [33,34,35]. Furthermore, CREB is activated downstream of multiple signaling pathways such as NMDAR-extracellular signal regulated kinases (ERK) and adenyl cyclase-protein kinase A (AC-PKA) cascades, that have been independently implicated in the maintenance of latent sensitization [36,37]. At the level of the DRt, we previously demonstrated that the AC–PKA cascade does not contribute to CREB activation [20], suggesting the involvement of alternative signaling pathways. In this context, the ERK pathway emerges as a plausible candidate. Notably, prolonged MOR stimulation has been shown to recruit β-arrestin, which not only promotes receptor desensitization but also functions as a signaling scaffold for MAPK/ERK pathways, thereby enhancing CREB phosphorylation [38,39]. Although β-arrestin signaling was not directly assessed in the present study, its engagement could plausibly contribute to the sustained elevation of pCREB and to long term molecular plasticity within the DRt following chronic morphine exposure. Importantly, β-arrestin–dependent mechanisms may act in parallel with, or in support of, persistent excitatory MOR signaling, thereby facilitating the maintenance of latent sensitization without being solely responsible for the expression of hyperalgesia.

Opioids can produce neuronal plasticity consistent with latent sensitization mechanisms at all levels of the nociceptive system. A substantial body of evidence has demonstrated that prior exposure to opioids enhances hyperalgesia produced by later administration of pronociceptive agents such as prostaglandin E2, through priming of primary afferent neurons, both in vivo and in vitro [17,18,19,40]. Spinal ascending nociceptive pathways are also affected by latent sensitization mechanisms since their blockade partially prevented the enhancement of hyperalgesia following surgical incision in rats previously exposed to fentanyl [12]. Importantly, priming of descending pathways by opioids seems to play a major role in latent sensitization. Descending pathways originating from the RVM are involved in mediating hyperalgesia during continuous morphine infusion [30] and inactivation of the rostroventral medial medulla (RVM) with lidocaine fully prevented the enhancement of post-operative hyperalgesia in rats previously exposed to fentanyl [12]. This brainstem area is involved in both descending pain inhibition and facilitation, with opioids playing a crucial role in modulating these opposing effects [1,21]. Furthermore, recent studies showed that morphine priming alters diffuse noxious inhibitory controls (DNIC), a paradigm that typically produces analgesia through normally functioning descending pain modulation [22,41]. Indeed, DNIC analgesia was lost after a seven-day morphine and recovered two weeks following morphine cessation [5]. In the same study, if the animals were injected with capsaicin following morphine cessation, DNIC analgesia remained absent two weeks following morphine cessation [5]. The exposition of rats to environmental stress also reinstated the loss of DNIC analgesia two weeks following morphine cessation [4]. It has been proposed that stress stimuli unmask enhanced descending facilitation thorough KOR signaling [4]. This is consistent with the broader understanding that loss of DNIC analgesia can result either from reduced descending pain inhibition or from increased descending pain facilitation modulation [22,27]. Altogether, these findings suggest that opioids can prime descending pain modulatory circuits, and that reactivation of these circuits is involved in reinstatement of hyperalgesia. Herein, we show that the persistent alteration of MOR signaling at the DRt represents an opioid-induced priming effect, through which enhanced facilitation can reinstate hyperalgesia.

We also evaluated the expression of MOR in the DRt during both the OIH and post-OIH phases. In contrast to pCREB, MOR expression in the DRt parallels the progression of nociceptive sensitivity, with elevated receptor levels during OIH and returning to baseline once hyperalgesia is resolved in the Post-OIH phase. The higher numbers of cells expressing MOR may result from altered receptor trafficking leading to an accumulation of MOR, as the canonical MOR-1 transcript remains unchanged during OIH [20]. This accumulation might be further enhanced by morphine, a poor internalizing agonist. Since the internalization of the receptor is necessary for targeting the receptor to degradation [42], impaired internalization could account for the increased cellular MOR protein levels observed during OIH. While MOR accumulation at the cell membrane likely contributes to the enhanced excitatory MOR signaling, the subsequent decrease in MOR levels in the Post-OIH phase may buffer the excitatory signaling of the receptor. Supporting this, we previously showed that either MOR knockdown or blockade of MOR-Gs coupling in the DRt significantly attenuated the development of OIH [20], and that inhibiting MOR-Gs coupling restored DAMGO-induced analgesia [20]. Thus, we posit that the reposition of MOR protein levels to baseline levels, in the Post-OIH phase, may help rebalance opioid-dependent signaling. In fact, opioid-dependent compensatory adaptations take place following the OIH phase, and these adaptations are responsible for the apparent normalization of nociceptive thresholds during this period. The best characterized compensatory mechanism is the induction of constitutive MOR activity. The pharmacological disruption of this constitutive signaling with inverse agonists such as naloxone or naltrexone reliably reinstates hypersensitivity, revealing a latent opioid-suppressed hyperalgesic state [14,15]. It has been proposed that the progressive normalization of nociceptive thresholds in the post-OIH phase relies on a dynamic balance between two opposing opioid-dependent processes, one analgesic and the other hyperalgesic [3,15]. It was also posited that this balance represents an allostatic rather than homeostatic state, leaving the nociceptive system sensitized and capable of reinstating hyperalgesia when allostasis is disrupted [3,15]. Of note, OIH has been increasingly linked to a six-transmembrane MOR isoform, which couples to excitatory Gs proteins [43,44], in contrast to the canonical seven-transmembrane MOR that preferentially couples to inhibitory Gi proteins [1]. Whether MOR-Gs coupling in the DRt is mediated by a six-transmembrane MOR isoform remains unknown, as the antibody used in our immunohistochemical assays does not distinguish between these two MOR isoforms. Nonetheless, the present study raises the possibility that two-opposing opioid-dependent mechanisms may also co-exist in the DRt. One mechanism may involve seven-transmembrane MOR-Gi signaling, whose activation inhibits descending pain facilitation during the period of normalization of nociceptive thresholds. The second mechanism may rely on MOR-Gs signaling, whose activation enhances descending facilitation and is likely a key contributor to the allostatic state established after opioid priming.

We used the rat plantar incision model [45], which reproduces both thermal and mechanical hypersensitivity, to evaluate the effects of opioid priming on post-operative hyperalgesia and on the efficacy of systemic morphine in treating post-operative hyperalgesia. This model was selected for its high clinical relevance; opioids remain the primary treatment for post-surgical pain [46], despite evidence that they often provide inadequate relief and can actively induce OIH [2,47]. Morphine, in particular, continues to be a cornerstone of post-operative analgesia [2], making it a critical target for investigating how prior opioid exposure alters subsequent therapeutic outcomes. Regarding the effect of opioid priming on post-operative hyperalgesia, we did not observe a significant enhancement of incision-induced hyperalgesia in animals previously exposed to morphine. This contrasts with other studies showing that opioids can potentiate and prolong hypersensitivity to subsequent injury [7,12]. Our results are probably due to the fact that we tested the animals shortly after the incision was performed, corresponding to an acute hyperalgesic phase, when any additional decreases of mechanical and thermal thresholds are unlikely to be detected by our behavioral assays. The additional effects of prior opioid treatment are more likely to be observed at later time-points as recovery from incision occurs. In line with that, the prior administration of fentanyl has been shown to enhance hypersensitivity 24 h after surgery for the induction of post-operative pain [12]. Similarly, increased hyperalgesia and allodynia were not observed in the incised hind paws of rats that had been pretreated with morphine within the first 24 h after incision [7,12].

Regarding the impact of opioid priming on the efficacy of systemic morphine, we observed a reduction in the antiallodynic potency of morphine in animals previously exposed to morphine. Curiously, its antihyperalgesic efficacy in the hot-plate test was preserved. This dissociation likely reflects the engagement of distinct neural circuits by mechanical versus thermal nociceptive modalities, as highlighted by recent studies [48,49]. Importantly, these circuits appear to exhibit differential sensitivity to opioid priming. For instance, peripheral MOR play an essential role in opioid-induced thermal and mechanical hyperalgesia [50,51] while MOR within descending brain-to-spinal opioid pathways preferentially regulate morphine-induced mechanical allodynia [52]. Morphine priming at the DRt likely contributes to enhance descending pain facilitation counteracting the inhibitory effects of opioids, and thereby contributing to a subsequent decrease in morphine efficacy. Supporting this interpretation, it has been demonstrated that the expression of MOR at supraspinal sites is determinant for the mediation of analgesia from systemic opioid drugs [53]. In particular, MOR within the DRt plays a critical role in mediating the analgesic actions of systemic morphine [26]. Thus, in the context of persistently altered MOR signaling at the DRt, morphine administration may favor MOR coupling to Gs proteins, destabilizing the allostatic state that temporarily stabilized descending pain facilitation, resulting in enhanced pain facilitation. Importantly, both inflammatory and neuropathic injuries produce sensitization processes that are additive to opioid-induced latent sensitization [14,15]. In this regard, we have shown that chronic inflammatory pain also shifts MOR signaling from inhibitory to excitatory at the DRt [27]. Furthermore, neuropathic pain induces a loss of inhibitory MOR function at the DRt through downregulation and desensitization of MOR [26]. Thus, the use of opioids to treat these conditions imposes a double burden on MOR signaling within descending pain facilitatory circuits. This further highlight the urgent need for adjuvant therapies capable of reducing the development of opioid-induced hyperalgesia and subsequent latent sensitization, especially in patients whose nociceptive circuits are already primed by injury.

## 4. Materials and Methods

### 4.1. Animals

All ethical guidelines for the study of experimental pain in lab animals as well as the European Communities Council Directive of 22 September 2010 (2010/63/EC) were followed. The experiments were approved by the Institutional Animal Care and Use Committee of the Faculty of Medicine of the University of Porto and the Portuguese National Authority for Animal Health (Ref. 0421/000/000). Adult male Wistar Han IGS rats (Charles River, Lyon, France) weighing 285 to 315 g were maintained under controlled temperature (22 ± 2 °C) and light (12/12 h light/dark cycle, lights on between 8:00 h and 20:00 h) conditions with ad libitum access to food and water. The animals were allowed to acclimate to the housing facility for at least one week before any procedure. All experiments were conducted during the light phase. The animals were housed in pairs, except following stereotaxic surgeries, after which they were housed individually to facilitate post-operative recovery.

### 4.2. Induction of OIH and Experimental Design

OIH was induced by morphine administered through either repeated subcutaneous (s.c.) injections or continuous infusion, for seven consecutive days. Following the seven-day treatment period with either method, the animals remained in the experimental protocols for an additional two-week period (i.e., until day 21).

*Induction of OIH by subcutaneous injections of morphine*: The animals received s.c. injections of morphine hydrochloride (10 mg/kg; Labesfal, Porto, Portugal), or saline, twice a daily (at 9 a.m. and 6 p.m.) for seven days as previously described [29]. A group of animals injected with saline (*n* = 7) and morphine (*n* = 4) was used for a time course evaluation of behavioral responses before and 30 min after the first injection of morphine (in the morning) on days 1, 3, 5, and 7 by the hot plate test. The von Frey test was also performed, in the same mornings, before any injection. The assessment of basal sensitivity, by these tests, continued on days 14 and 21, i.e., one and two weeks after treatment cessation, respectively.

*Induction of OIH by continuous infusion of morphine*: Morphine hydrochloride (generously provided by Dr. Paulo Cruz, Porto Military Hospital, Porto, Portugal) was infused at a rate of 45 μg μL^−1^·h^−1^ using ALZET^®^ osmotic minipumps (model 2001; DURECT Corporation, Cupertino, CA, USA) for seven days, as previously described [20]. Control animals were infused with the saline vehicle solution. The minipumps were implanted under isoflurane anesthesia. The dorsum of the animals was shaved and cleaned with Betadine^®^ and a midline incision was made in the rat dorsum for the introduction of the mini-pumps. At the end of the procedure the skin was closed with surgical staples (Stoelting Co., Wood Dale, IL, USA) and the animals returned to their cage. The animals were monitored daily to evaluate body weight and to detect signs caused by incorrect functioning of the mini-pumps such as teeth chattering, diarrhea, rhinorrhea, ptosis, irritability, lacrimation, escaping, penile erection, or abnormal posture [54]. The minipumps were removed after seven days, under isoflurane anesthesia. One group of animals (saline-infused *n* = 6 and morphine-infused *n* = 5) was used for the time course assessment of basal sensitivity. Three additional experimental groups, prepared as described above, were used for specific purposes detailed in the following sections: (i) DAMGO assays; (ii) induction of post-operative pain; and (iii) immunohistochemical assays.

### 4.3. DAMGO Assays

*Stereotaxic Surgeries*: Stereotaxic surgeries were performed for the implantation of a guide cannula above the left DRt. The procedure was performed one week after removal of minipumps (terminating the infusion of saline *n* = 4 and morphine *n* = 7). For that, the animals were placed in a stereotaxic frame, under deep anesthesia with an intraperitoneal mixture of ketamine hydrochloride (0.06 g/Kg) and medetomidine (0.25 g/Kg), and the following the coordinates of the atlas Paxinos and Watson 2004 [55] relative to the interaural line were used. Anterior–Posterior: −6.0 mm; Medial-Lateral: −1.4 mm; Dorsal–Ventral: −1.5 mm.

*Microinjection of DAMGO*: DAMGO was microinjected through the guide cannula, implanted above the left DRt, one week after the stereotaxic surgery (corresponding to two weeks after termination of saline or morphine infusion). The animals were injected with 0.1 ng of DAMGO dissolved in saline, using a stainless-steel needle protruding 3 mm beyond the cannula. A volume of 0.5 μL was infused over a period of 1 min. The saline vehicle solution was injected at the DRt of saline-infused animals (*n* = 6). All tests were conducted by an experimenter blinded to the treatments. The effects of DAMGO and saline injections were tested by the von Frey and hot plate tests conducted before and 15 min after injection. The dose and timing of DAMGO action were chosen based on previous studies performed at the DRt [20,26,27].

### 4.4. Induction of the Post-Operative Pain

The model was induced two weeks after termination of morphine- or saline-infusion (*n* = 7 each), following the procedures previously described [45]. Briefly, under isoflurane, the plantar aspect of the left hindpaw was cleaned with Betadine (iNova Pharmaceuticals, Singapore). A 1 cm longitudinal incision was made with a blade, through the skin and fascia of the plantar aspect of the foot, starting 0.5 cm from the proximal edge of the heel and extending toward the toes. The plantaris muscle was then elevated and incised longitudinally. At the end of the procedure, the skin was closed with two mattress sutures.

### 4.5. Morphine Dose-Response Experiments

To evaluate the analgesic potency of systemic opioids in post-operative pain, morphine was administered immediately after induction of post-operative pain. The animals received first an injection of saline (s.c.), followed by incrementing doses of morphine s.c. (0.1; 0.4; 1; 4; 10 mg/kg). The von Frey and the hot-plate tests, were conducted before and after post-operative pain induction and also 30 min after each dose of morphine at its peak of action [56]. Each dose of morphine was administered every 30 min immediately after testing the previous dose. Data was converted to percent maximum possible effect (%MPE) according to the equation: % MPE = (Post drug value − Pre-drug value)/(Ceiling value − Pre-drug value) × 100. In the von Frey test, the ceiling value (i.e., the maximum stimulus applied after morphine injection) was 100 g in saline-infused animals, and 180 g in morphine-infused. Paw withdrawal thresholds obtained in the von Frey test were log transformed for the estimation of MPE. To determine the %MPE from the data obtained in the hot-plate test, a cutoff latency of 30 s was taken as the ceiling value. %MPE values are presented as mean ± SD. Dose-response curves were plotted as %MPE vs. dose and fitted with nonlinear regression (variable slope model) to determine ED50 values with 95% confidence intervals (GraphPad Prism v7).

### 4.6. Nociceptive Behavior

The animals were habituated to the experimenter and the experimental environment for a period of one week. The von Frey test was performed by placing the animals on an elevated transparent cage with a mesh wire bottom allowing the stimulation of the plantar surface of the left hind paw with calibrated von Frey monofilaments (Stoelting Co., Wood Dale, IL, USA) with logarithmically incremental stiffness ranging from 0.4 g to 60 g. Testing started with the 2-g filament applied perpendicularly to the plantar surface for 3 s. Withdrawal thresholds were determined using the Dixon up-and-down method [57]. The hot plate test was performed by placing the animals on a hotplate system (BIOCHP Cold Hot Plate Test, Bioseb SAS, Vitrolles, France), with a surface temperature of 52 °C. The nociceptive threshold was quantified as the latency (in seconds) to licking, retraction of the hind paw, or jumping after placement of the rat on the hotplate. A 30-s cut off was used to avoid tissue damage.

### 4.7. Tissue Preparation and Immunohistochemistry

Immunohistochemical assays were performed on two experimental groups: (i) a group of animals, hereafter denominated OIH group, which received a 7-day infusion of saline and morphine (*n* = 6 each) and was euthanized immediately after the infusion period; and (ii) a group, hereafter denominated Post-OIH group, which was infused with saline or and morphine (*n* = 6 each) for 7 days, but were euthanized on day 21 (corresponding to two weeks after infusion termination). The animals were deeply anesthetized with an overdose of sodium pentobarbital (70 mg/kg intraperitoneal) and perfused through the ascending aorta with 100 mL of calcium free Tyrode’s solution, followed by 750 mL of a fixative solution containing 4% paraformaldehyde in 0.1 M phosphate buffer, pH 7.2. The brainstems were removed, immersed in fixative for 4 h followed by 30% sucrose in 0.1 M phosphate- buffered saline overnight, at 4 °C, and sliced at 40 μm in coronal orientation in a freezing microtome. One in every fourth section encompassing the dorsal reticular nucleus was used for the immunodetection of MOR and pCREB.

***Immunoreactions:*** Before incubation with the primary antibodies, tissue sections were treated in 0.1 M phosphate-buffered saline (PBS) containing 30% hydrogen peroxide (H_2_O_2_) to block endogenous peroxidase activity. This was followed by a 2-h incubation in a blocking solution composed of 0.1 M glycine and 10% normal swine serum (NSS) diluted in PBS-T (PBS supplemented with 0.3% Triton X-100), to prevent nonspecific antibody binding. The sections were then incubated with the following primary antibodies diluted in PBS-T containing 2% NSS: (1) a rabbit polyclonal antibody against MOR (Neuromics, Inc., Edina, MN, USA), diluted at 1:1000 for 48 h, at 4 °C; and (2) a rabbit polyclonal antibody against pCREB (Millipore Corporation, Bedford, MA, USA), diluted at 1:1000 for 48 h, at 4 °C. The sections were washed in PBS-T in 2% NSS and incubated with a biotinylated horse anti-rabbit secondary antibody (at 1:200 for 1 h; Vector Laboratories, Inc., Burlingame, CA, USA) diluted in PBS-T with 2% NSS. After several washes with PBS-T, the sections were incubated in avidin-biotin complex (ABC; 1/200 in PBST; Vector Laboratories, Burlingame, CA, USA) for 1 h. After washing with PBS-T, PBS, and Tris-HCl pH 7.6 (0.05 M), diaminobenzidine (DAB) was used to reveal the labeling and the sections were mounted on gelatin-coated slides.

The specificity of the anti–MOR antibody was previously tested by blocking the antibody with a blocking peptide in immunohistochemistry and western blot analysis [58]. We further tested each antibody specificity by performing negative controls with omission of either the primary or the secondary antibodies which blocked all the immunostaining.

***Quantification of immunolabelling:*** The quantification of each protein was performed in five sections encompassing the rostrocaudal extent of the DRt, randomly taken from each rat, by an experimenter blinded as to the experimental group. The DRt was localized and delimited according to the Paxinos and Watson Rat Brain Atlas [55].

For the quantification of MOR, a light microscope (microscope Axioskop 40 model, Zeiss^®^, Hombrechtikon, Switzerland) was used to manually count the numbers of MOR-immunoreactive (IR) neurons in the left and right DRt, using the 20× objective, by an experimenter blinded as to the experimental group. The results were presented as a mean ± SD of the number of MOR-IR cells per section.

For the quantification of pCREB, a light microscope (microscope Axioskop 40 model, Zeiss^®^, Hombrechtikon, Switzerland) was used to capture photomicrographs of pCREB labelling with a Zeiss Axiocam 208 color camera (Zeiss^®^, Hombrechtikon, Switzerland) and the the Zen 3.3 software (Zeiss^®^, Hombrechtikon, Switzerland). The number of pCREB positive nuclei in the left and right DRt was determined using an automated cell counter plugin of the ImageJ software version 1.52u (US National Institutes of Health, Bethesda, MD, USA, free access). The results were presented as mean ± SD of the total number of pCREB^+^ nuclei.

### 4.8. Histology

After the last behavioral evaluation, the animals used for DAMGO assays were administered 0.5 μL of 0.6% Chicago sky blue dye (Sigma-Aldrich Química S.A., Lisbon, Portugal) through the guide cannula and euthanized by an overdose of sodium pentobarbital. Only injection sites encompassing the DRt were included for analysis.

### 4.9. Statistical Analysis

Two-way mixed ANOVA for repeated measurements was used for the analysis of behavioral data presented in Figure 1, Figure 2, Figure 3, Figure 6C,D and Figure 7. In case of a significant interaction with proceeded with multiple comparisons. Tukey’s post hoc test was applied for Figure 1 and Figure 3, whereas Bonferroni’s test was used for pairwise comparisons between pre- and post-injection time points in Figure 2 and Figure 6C,D. One-way repeated-measures ANOVA was used for the analysis of behavioral data presented in Figure 6A,B followed by multiple comparisons using Tukey’s post hoc test. Mechanical threshold responses, obtained in the von Frey test, were logarithmic transformed to enable ANOVA analysis. The expression of MOR and pCREB (Figure 4 and Figure 5) both the OIH and Post-OIH groups were analyzed by unpaired t-tests. The normality assumption was checked by inspection of the distribution of the variables both with q-q plots and histograms. However, we must acknowledge that the sample size limits the ability to detect departures from normality. The statistical analysis was performed by GraphPad Prism v7 and SPSS v24. The significance level was set at 0.05 and all statistical tests were two-tailed.

## Data Availability

The original contributions presented in this study are included in the article. Further inquiries can be directed to the corresponding author.
